# Molecular characterization of breast cancer: a potential novel immune-related lncRNAs signature

**DOI:** 10.1186/s12967-020-02578-4

**Published:** 2020-11-07

**Authors:** Jianguo Lai, Bo Chen, Guochun Zhang, Xuerui Li, Hsiaopei Mok, Ning Liao

**Affiliations:** Department of Breast Cancer, Cancer Center, Guangdong Provincial People’s Hospital, Guangdong Academy of Medical Sciences, 106 Zhongshan Er Road, Yuexiu district, Guangzhou, 510080 Guangdong China

**Keywords:** Breast cancer, Immune, lncRNA, Signature, Survival

## Abstract

**Background:**

Accumulating evidence has demonstrated that immune-related lncRNAs (IRLs) are commonly aberrantly expressed in breast cancer (BC). Thus, we aimed to establish an IRL-based tool to improve prognosis prediction in BC patients.

**Methods:**

We obtained IRL expression profiles in large BC cohorts (N = 911) from The Cancer Genome Atlas (TCGA) database. Then, in light of the correlation between each IRL and recurrence-free survival (RFS), we screened prognostic IRL signatures to construct a novel RFS nomogram via a Cox regression model. Subsequently, the performance of the IRL-based model was evaluated through discrimination, calibration ability, risk stratification ability and decision curve analysis (DCA).

**Results:**

A total of 52 IRLs were obtained from TCGA. Based on multivariate Cox regression analyses, four IRLs (A1BG-AS1, AC004477.3, AC004585.1 and AC004854.2) and two risk parameters (tumor subtype and TNM stage) were utilized as independent indicators to develop a novel prognostic model. In terms of predictive accuracy, the IRL-based model was distinctly superior to the TNM staging system (AUC: 0.728 VS 0.673, *P* = 0.010). DCA indicated that our nomogram had favorable clinical practicability. In addition, risk stratification analysis showed that the IRL-based tool efficiently divided BC patients into high- and low-risk groups (*P* < 0.001).

**Conclusions:**

A novel IRL-based model was constructed to predict the risk of 5-year RFS in BC. Our model can improve the predictive power of the TNM staging system and identify high-risk patients with tumor recurrence to implement more appropriate treatment strategies.

## Background

Globally, breast cancer (BC) is the most frequently common carcinoma in women [1, 2]. In light of the statistics of the American Cancer Society, approximately 279,100 new BC cases and 42,690 cancer deaths are estimated to occur in the United States in 2020 [3]. Although various therapeutic strategies are currently being applied to improve BC prognosis, including surgery, chemotherapy, radiotherapy, endocrine therapy and targeted therapy, many BC patients still have poor survival outcomes because of recurrence [4]. Survival prognosis prediction is mainly based on the American Joint Committee on Cancer (AJCC) tumor-node-metastasis (TNM) staging system. However, it should be noted that BC patients at the same TNM stage may have distinct survival outcomes [5]. This highlights that the TNM staging system is inadequate to achieve accurate prognosis evaluation and that it is unable to reflect the biological heterogeneity of BC. For example, the system is mainly based on clinicopathological parameters rather than on molecular signatures [6, 7]. Increasing evidence has revealed that molecular biomarkers have the potential to improve prognostic assessment and identify high-risk cancer patients [8–14]. Accordingly, there is an urgent need to screen effective molecular biomarkers for improving survival prognosis prediction and identify high-risk BC patients with tumor recurrence.

With advancements in transcriptomics, long noncoding RNAs (lncRNAs) have captured considerable attention in human cancers in the past decade [15–19]. LncRNAs are a class of mRNA-like transcripts with a length of > 200 nucleotides that lack protein-coding ability. Accumulating evidence indicates that lncRNAs play important roles in various biological processes, including transcriptional modifications, cell proliferation, differentiation, epigenetic modulation, and immune system-modulated pathways [20–25]. For example, lncRNA SNHG1 enhances the differentiation of Treg cells to provoke immune escape in BC [26]. LncRNA AC025580.2 has a positive influence on immune escape in pancreatic cancer [27]. In addition, a large number of lncRNAs have been confirmed to act as diagnostic and prognostic biomarkers or potential therapeutic targets in multiple tumors [11, 28–32]. However, the role of immune-related lncRNAs (IRLs) in the prognostic evaluation of BC remains unclear.

Hence, the objective of this study was to establish and validate a novel IRL model to predict the 5-year RFS of BC patients. This tool is able to improve the predictive accuracy of the TNM staging system and identify high-risk BC patients with tumor recurrence to facilitate optimal therapeutic schemes.

## Methods

### Patients and study design

Gene and lncRNA expression profiles as well as corresponding BC cases information were acquired from The Cancer Genome Atlas (TCGA) database. The inclusion criteria were as follows: (1) pathologically confirmed invasive BC, (2) complete follow-up data, including RFS status and survival time, and (3) survival time > 1 month. The following clinical parameters for each patient were collected: age, estrogen receptor (ER) status, progesterone receptor (PR) status, T stage, N stage, TNM stage, human epithelial growth factor receptor 2 (HER2) status, tumor subtype, RFS, and survival time. The Research Ethics Committee of Guangdong Provincial People’s Hospital (GDPH) approved our study. As the study used data from the database TCGA, written informed consent was waived.

### Establishment of IRL risk score and the IRL-based model

First, immune-related genes (IRGs) were retrieved from the Molecular Signatures Database (MSDB) v4.0 (immune response M19817, and immune system process M13664) [33, 34]. According to the GENCODE project, lncRNA expression data were obtained from the gene expression profile [35]. Next, IRLs were defined based on association analysis between the mRNA expression level and lncRNA expression data (|R|> 0.7, *P* < 0.05). Subsequently, univariate Cox proportional hazards regression analysis (CPHRA) was conducted to explore the association between the IRL expression level and patient RFS. Multivariate CPHRA was carried out to investigate independent prognostic IRLs. Regarding multivariate analysis, the collinearity diagnosis was confirmed via the variance inflation factor (VIF), and the final independent prognostic IRLs were selected to develop the IRL-based risk score, as follows: IRL risk score = sum of coefficients × expression level of IRLs. Finally, a novel IRL-based model incorporating the IRL signature and clinical variables was constructed to improve the prediction power of TNM staging in BC patients.

### Assessment of the IRL-based nomogram

The area under the curve (AUC) of the time-dependent receiver operating characteristic (ROC) curve for 5-year RFS was calculated to evaluate the performance of the IRL-based model. The nomogram score for each patient was allocated on the basis of the IRL-based nomogram. Patients were divided into low- and high-risk nomogram score groups using the median score as the cutoff point. Subgroup analysis was applied to assess the risk stratification ability of the IRL-based model. A calibration curve was drawn to estimate the calibration ability of the IRL-based nomogram. Decision curve analysis (DCA) was used to weigh the clinical practicability of the IRL-based nomogram [36–41].

### Gene enrichment analysis of the IRL signature

Gene enrichment analysis of the four IRLs was applied using Metascape, a free online method for gene annotation [42]. Functional pathway enrichment was assessed on the basis of co-expressing genes of the four IRLs in the same module.

### Statistical analysis

The χ^2^ test, Fisher's exact test or Mann–Whitney U test was employed to explore differences in indicators. The optimal cut-off values of IRL-based nomogram scores were calculated via X-tile software, version 3.6.1 (Yale University, New Haven, CT, USA). Kaplan–Meier survival analysis and the log-rank test were used to examine the 5-year RFS difference between low- and high-risk groups. All statistical analyses were utilized via R software (www.r-project.org, version 3.6.1) and Stata/MP, version 14.0 (StataCorp LP, College Station, TX). A P value < 0.05 was defined as significant.

## Results

### Baseline characteristics

In total, 911 BC samples were collected as the primary cohort in our study. As in our previous studies, each patient can be assigned a computer-generated allocation number (0 or 1) based on Stata software, and two cohorts were differentiated from the primary cohort according to the allocation number. Of the total, 456 patients (allocation number = 0) were considered as the validation cohort [4, 5]. The baseline characteristics of all patients in the two datasets are shown in Table 1. There were no significant differences between the variables examined in the two independent cohorts (all *P* > 0.05). The median age of the included patients was 58 years (IQR: 48–66) and 58 years (IQR: 48–65) in the two datasets.

**Table 1:** Baseline characteristics of the included patients

Variables	Primary cohort	Validation cohort	*P* value
	No. (%)	No. (%)	
No. of patients	911	456	
Age (years)	58 (48, 66)	57 (48, 67)	0.862
T stage			0.699
T1	248 (27.2)	125 (27.4)	
T2	529 (58.1)	254 (55.7)	
T3	110 (12.1)	65 (14.3)	
T4	24 (2.6)	12 (2.6)	
N stage			0.266
N0	435 (47.8)	224 (49.1)	
N1	306 (33.6)	150 (32.9)	
N2	95 (10.4)	48 (10.5)	
N3	65 (7.1)	34 (7.5)	
Nx	10 (1.1)	0 (0)	
TNM stage			0.878
I	161 (17.7)	82 (18.0)	
II	527 (57.8)	261 (57.2)	
III	206 (22.6)	107 (23.5)	
Unknown	17 (1.9)	6 (1.3)	
ER status			0.723
Negative	192 (21.1)	90 (19.7)	
Positive	682 (74.8)	350 (76.8)	
Unknown	37 (4.1)	16 (3.5)	
PR status			0.920
Negative	275 (30.2)	137 (30.0)	
Positive	598 (65.6)	302 (66.2)	
Unknown	38 (4.2)	17 (3.7)	
HER2 status			0.265
Negative	653 (71.7)	340 (74.6)	
Positive	155 (17.0)	62 (13.6)	
Unknown	103 (11.3)	54 (11.8)	
Molecular subtype			0.495
HR+/HER2-	523 (57.4)	277 (60.7)	
HR+/HER2 +	124 (13.6)	52 (11.4)	
HR−/HER2+	31 (3.4)	10 (2.2)	
TNBC	129 (14.2)	62 (13.6)	
Unknown	104 (11.4)	55 (12.1)	

*TNM *tumor-node-metastasis, *ER* estrogen receptor, *PR* progesterone receptor, *HER2* human epithelial growth factor receptor 2

### Identification of the IRL signature

First, 332 IRGs were collected from Molecular Signatures Database (MSDB), and the expression profiles of the 332 IRGs were downloaded from TCGA. We defined a lncRNA with (|R|> 0.7 and P < 0.05) expression levels between the lncRNAs and genes as IRLs. Thus, on the basis of Pearson correlation analysis, 52 lncRNAs were confirmed as IRLs (|R|> 0.7 and P < 0.05). After univariate CPHRA, 4 IRLs were identified in the primary cohort (*P* < 0.05). Subsequent multivariate CPHRA further screened four IRLs (A1BG-AS1, AC004477.3, AC004585.1 and AC004854.2) as independent prognostic factors in the primary cohort. The VIFs ranged from 1.01 to 1.03, indicating no collinearity among the predictors. The detailed characteristics of the four IRLs in the training dataset are listed in Table 2.

**Table 2:** The four-IRLs significantly associated with 5-year RFS in the primary cohort

Ensemble ID	Gene name	Genomic location	HR	P value	Coefficient
ENSG00000268895	A1BG-AS1	chr 19: 58,347,718–58,355,455	0.637	0.013	− 0.451
ENSG00000278765	AC004477.3	chr 17: 48,066,704–48,067,293	1.341	0.007	0.294
ENSG00000266088	AC004585.1	chr 17: 40,516,892–40,527,002	0.637	0.005	− 0.450
ENSG00000272768	AC004854.2	chr 7: 44,884,953–44,886,393	1.873	0.001	0.627

*RFS* relapse-free survival

### Development of the IRL-based risk score and prognostic nomogram

**Fig. 1:**
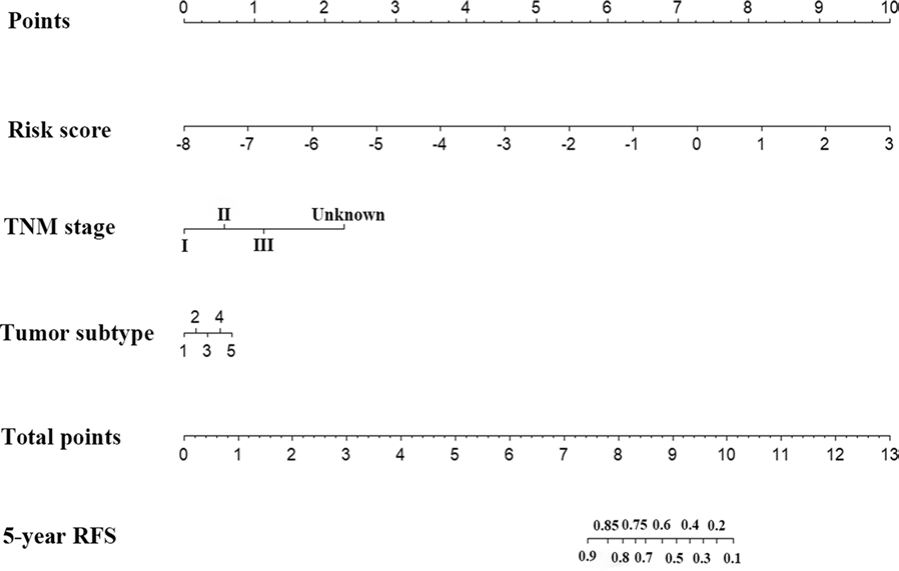
The immune-related lncRNAs model to predict 5-year RFS in breast cancer patients. 1 HR+/HER2-, 2 HR+/HER2+, 3 HR-/HER2+, 4 TNBC, and 5 unknown

An IRL-based risk score formula was built on the basis of IRL expression levels and their estimated regression coefficients in the multivariate CPHRA, where IRL-based risk score = −0.451 × expression_A1BG-AS1_ + 0.294 × expression_AC004477.3_–0.450 × expression_AC004585.1_ + 0.627 × expression_AC004854.2_. Based on univariate and multivariate CPHRA (Table 3), three prognostic variables (IRL-based risk score, tumor subtype, and TNM stage) were selected as independent indicators to build an IRL-based model. The novel IRL-based nomogram is illustrated in Fig. 1.

**Table 3:** Univariate and multivariate analyses in the primary cohort

Variables	Univariate analysis		Multivariate analysis	
	HR (95% CI)	*P* value	HR (95% CI)	*P* value
Age	1.002 (0.986–1.018)	0.853		
T stage				
T1	Referent			
T2	1.631 (0.980–2.714)	0.060		
T3	2.027 (1.060–3.876)	*0.033*		
T4	5.748 (2.394–13.805)	* < 0.001*		
N stage				
N0	Referent			
N1	1.611 (1.004–2.585)	*0.048*		
N2	2.282 (1.219–4.269)	*0.010*		
N3	4.651 (2.425–8.921)	* < 0.001*		
Nx	15.987 (6.600–38.722)	* < 0.001*		
TNM stage				
I	Referent		Referent	
II	1.641 (0.850–3.167)	0.140	1.589 (0.820–3.077	0.170
III	3.589 (1.824–7.061)	*< 0.001*	4.015 (2.010–8.020)	*< 0.001*
Unknown	13.559 (5.415–33.951)	*< 0.001*	12.054 (4.623–31.430)	*< 0.001*
ER status				
Negative	Referent			
Positive	0.562 (0.369–0.855)	*0.007*		
Unknown	1.097 (0.426–2.825)	0.848		
PR status				
Negative	Referent			
Positive	0.550 (0.369–0.821)	*0.003*		
Unknown	1.120 (0.442–2.838)	0.811		
Tumor subtype				
HR+/HER2−	Referent		Referent	
HR+/HER2+	0.756 (0.339–1.687)	0.495	0.792 (0.354–1.774)	0.571
HR−/HER2+	1.744 (0.624–4.872)	0.289	0.924 (0.320–2.672)	0.884
TNBC	2.038 (1.204–3.450)	*0.008*	2.318 (1.359–3.955)	*0.002*
Unknown	2.591 (1.572–4.269)	* < 0.001*	1.922 (1.153–3.204)	*0.012*
Risk score	2.718(1.903–3.884)	* < 0.001*	2.975 (2.028–4.364)	* < 0.001*

*CI* confidence interval, *HR* hazard ratios, *ER* estrogen receptor, *PR* progesterone receptor, *HER2*, human epithelial growth factor receptor 2

Italic values indicate statistical significance (*P* < 0.05)

### Evaluation of the IRL-based prognostic nomogram

**Fig. 2:**
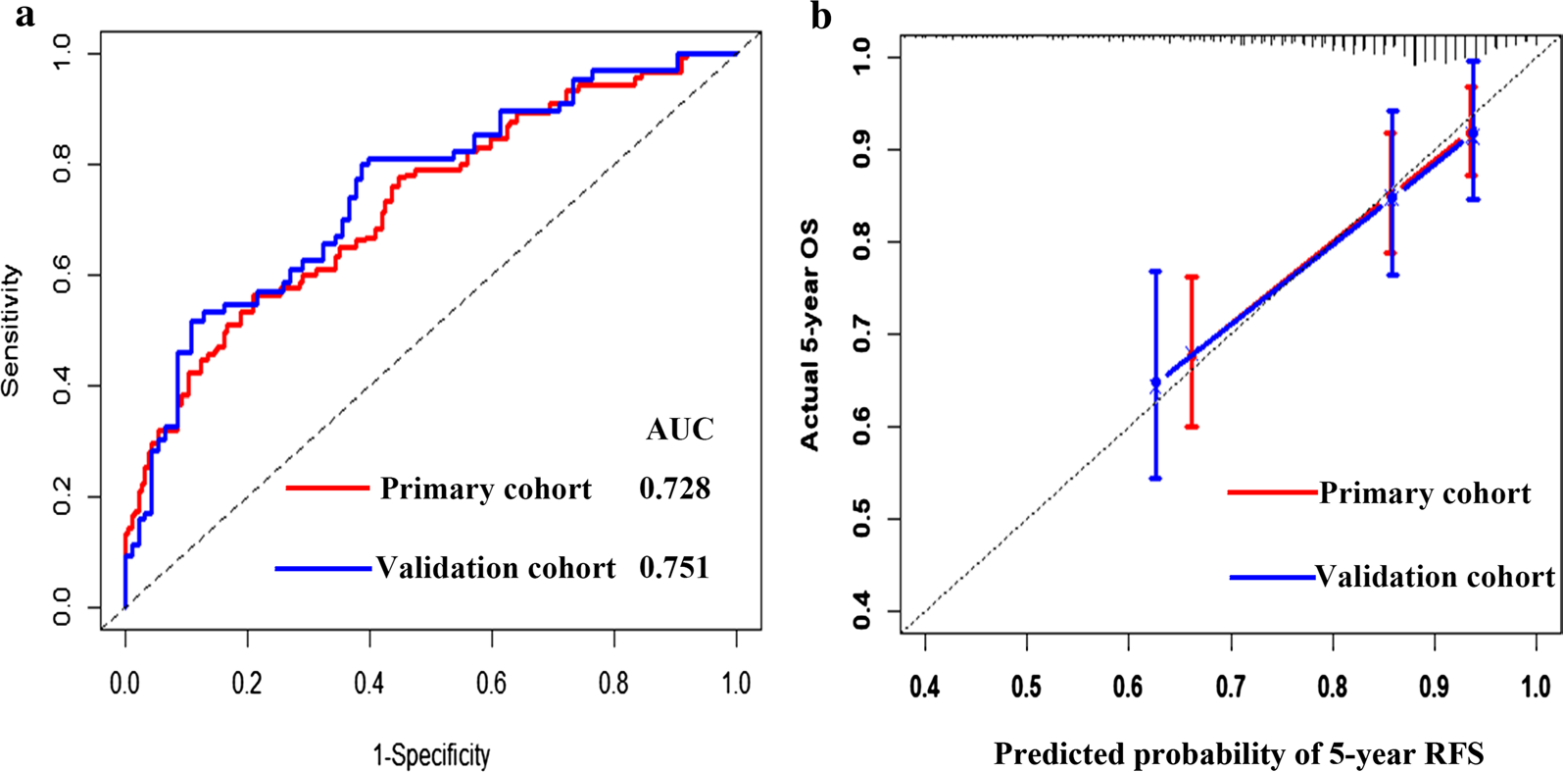
**a** Time-dependent receiver operating characteristic (ROC) curves at 5-years based on the immune-related lncRNAs model in the primary cohort and validation cohort. **b** Calibration curves of the immune-related lncRNAs model in the primary cohort and validation cohort

**Fig. 3:**
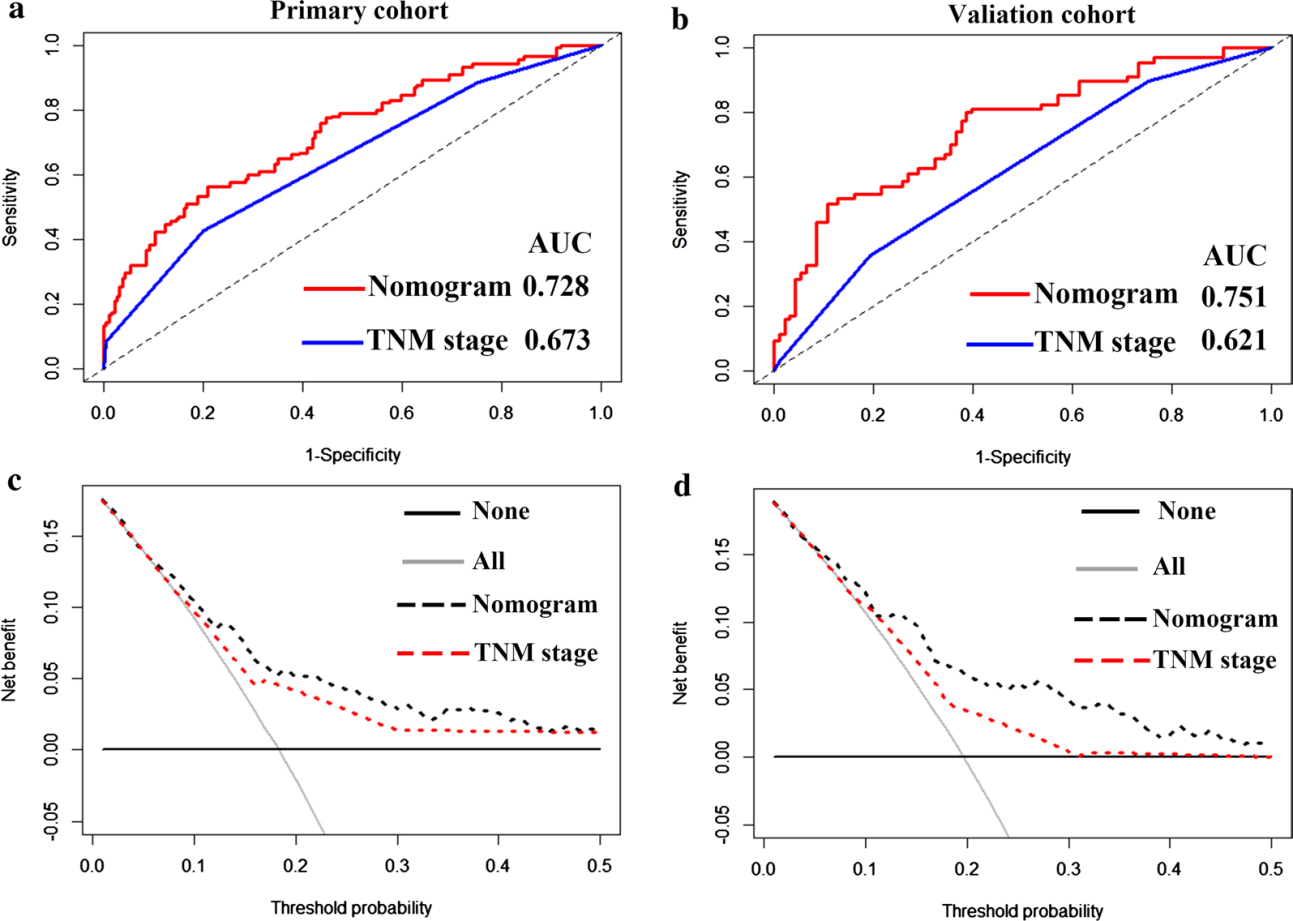
Comparisons of the predictive accuracy between the immune-related lncRNAs model and TNM stage using time-dependent ROC curves in the primary cohort (**a**) and validation cohort (**b**). Comparisons of the clinical utility between the immune-related lncRNAs model and TNM stage using decision curve in the primary cohort (**c**) and validation cohort (**d**)

**Fig. 4:**
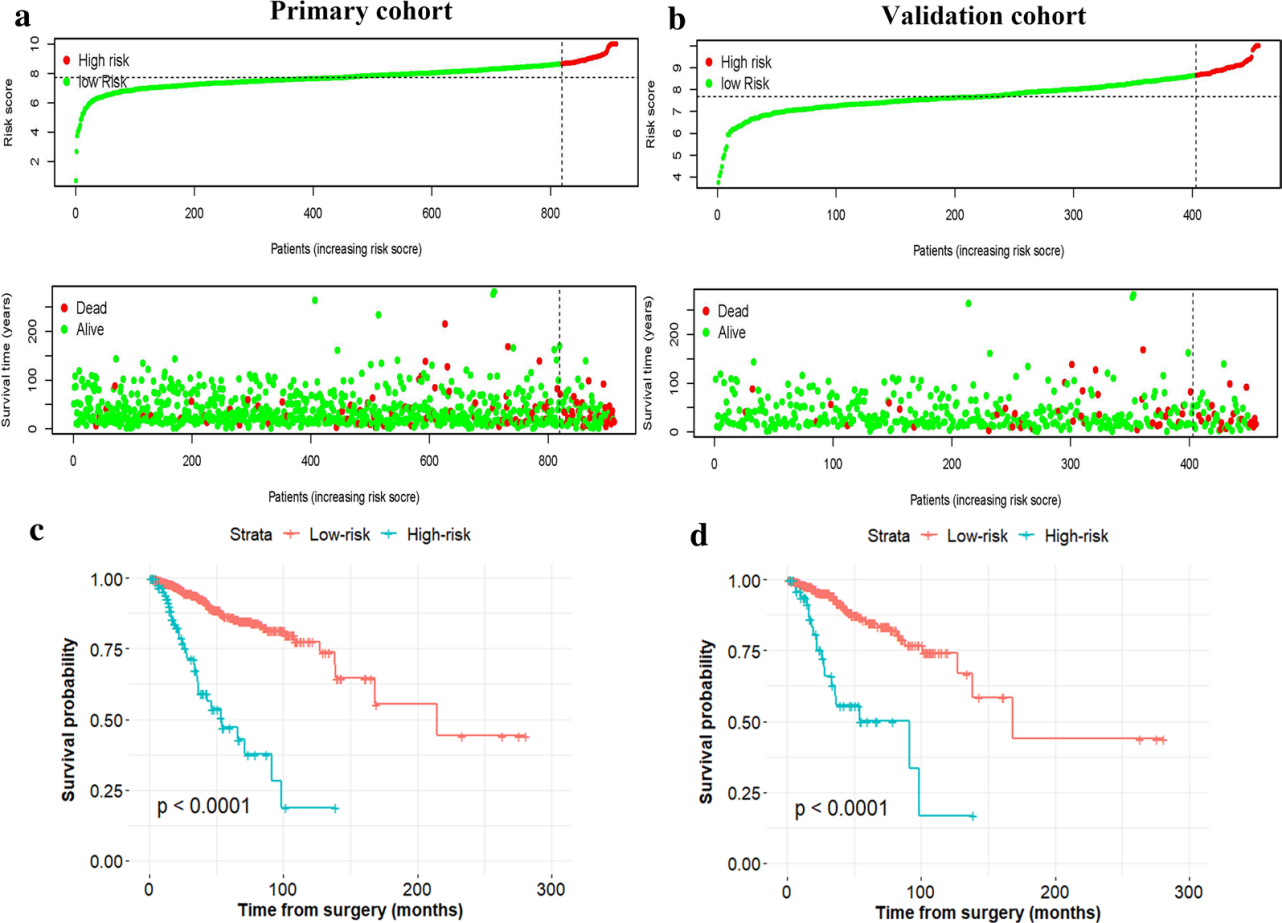
The distribution of immune-related lncRNAs model score, RFS, and RFS status in the primary cohort (**a**) and validation cohort (**b**). The dotted line indicates the optimal cutoff value of the model score to divide patients into the low- and high-risk set. Kaplan–Meier curves of the low- and high-risk patients based on the immune-related lncRNAs model in the primary cohort (**c**) and validation cohort (**d**)

**Fig. 5:**
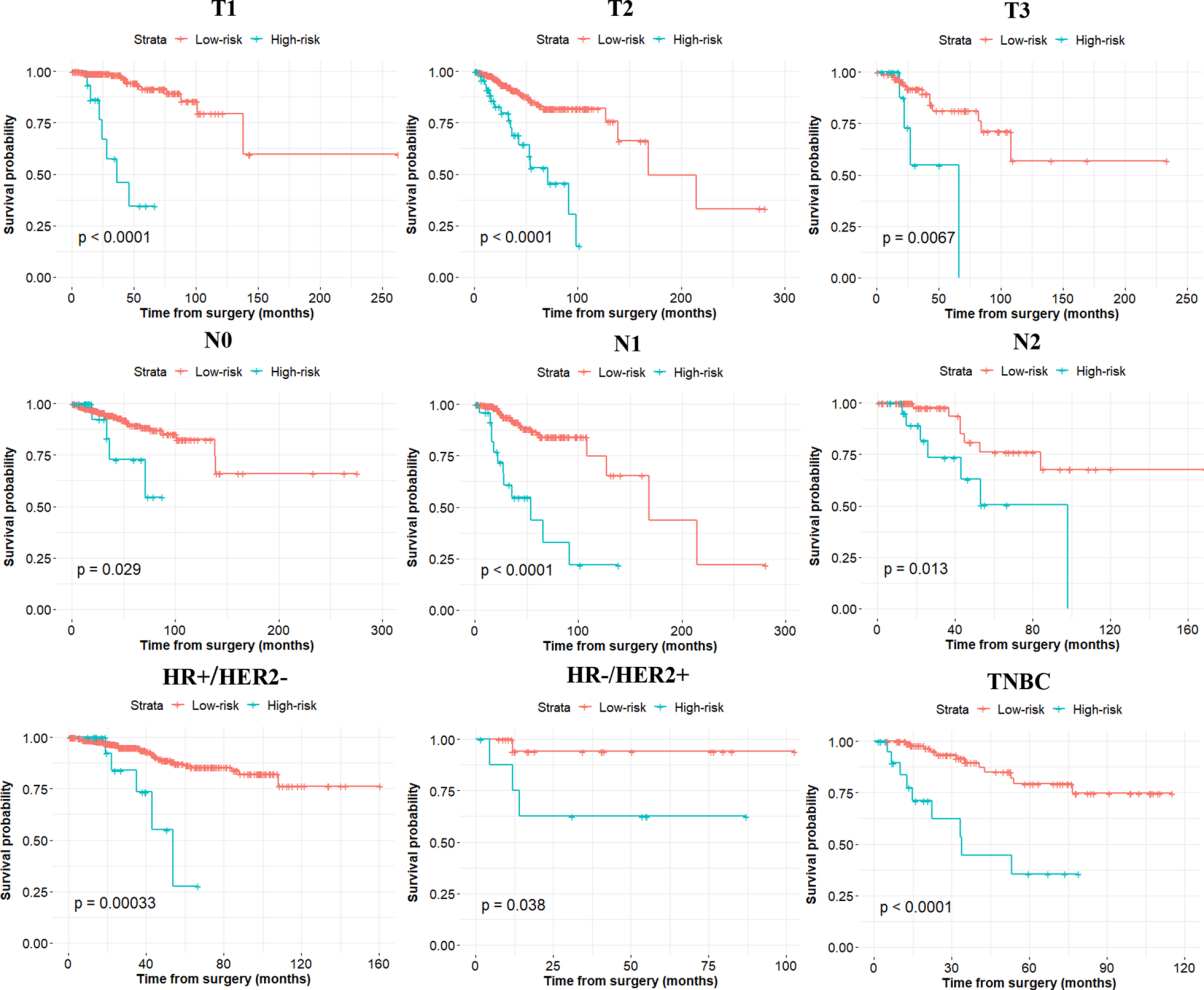
Subgroup analysis of the immune-related lncRNAs model for breast cancer patients in T stage, N stage, and tumor subtype

The AUCs of the IRL-based nomogram were 0.728 (95% CI: 0.658–0.797) and 0.751 (95% CI: 0.656–0.846) in the primary and validation datasets, respectively, showing that this model had good predictive performance (Fig. 2a). Calibration plots revealed ideal agreement between the IRL-based nomogram-predicted likelihoods and the actual observations of 5-year RFS, indicating that this tool had high calibration ability (Fig. 2b). In addition, compared with the predictive accuracy between the IRL-based model and TMN stage, the time-dependent ROC curve suggested that the IRL-based nomogram outperformed the TNM stage for both the primary and validation cohorts (*P* < 0.05, Fig. 3a, b). Moreover, DCA indicated that the IRL-based tool added more net benefit than did the TNM stage for the primary and validation datasets; therefore, this nomogram showed superior clinical usefulness (Fig. 3c, d). On the basis of the IRL-based model, patients in the primary and validation cohorts were stratified into low- or high-risk groups using the median nomogram score as the cutoff point. The distribution characteristics of the IRL-based model score, RFS, and RFS status are shown in Fig. 4, indicating that patients with lower nomogram scores had better 5-year RFS than those with higher nomogram scores (*P* < 0.001). Subgroup analyses demonstrated that the IRL-based model had good risk stratification ability for T1 (*P* < 0.0001), T2 (*P* < 0.0001), T3 (*P* = 0.0067), N0 (*P* = 0.029), N1 (*P* < 0.0001), N2 (*P* = 0.013), HR + /HER2- (*P* = 0.00033), HR-/HER2 + (*P* = 0.038), and TNBC (*P* < 0.0001) (Fig. 5).

### Functional enrichment analysis of the IRL signature

**Fig. 6:**
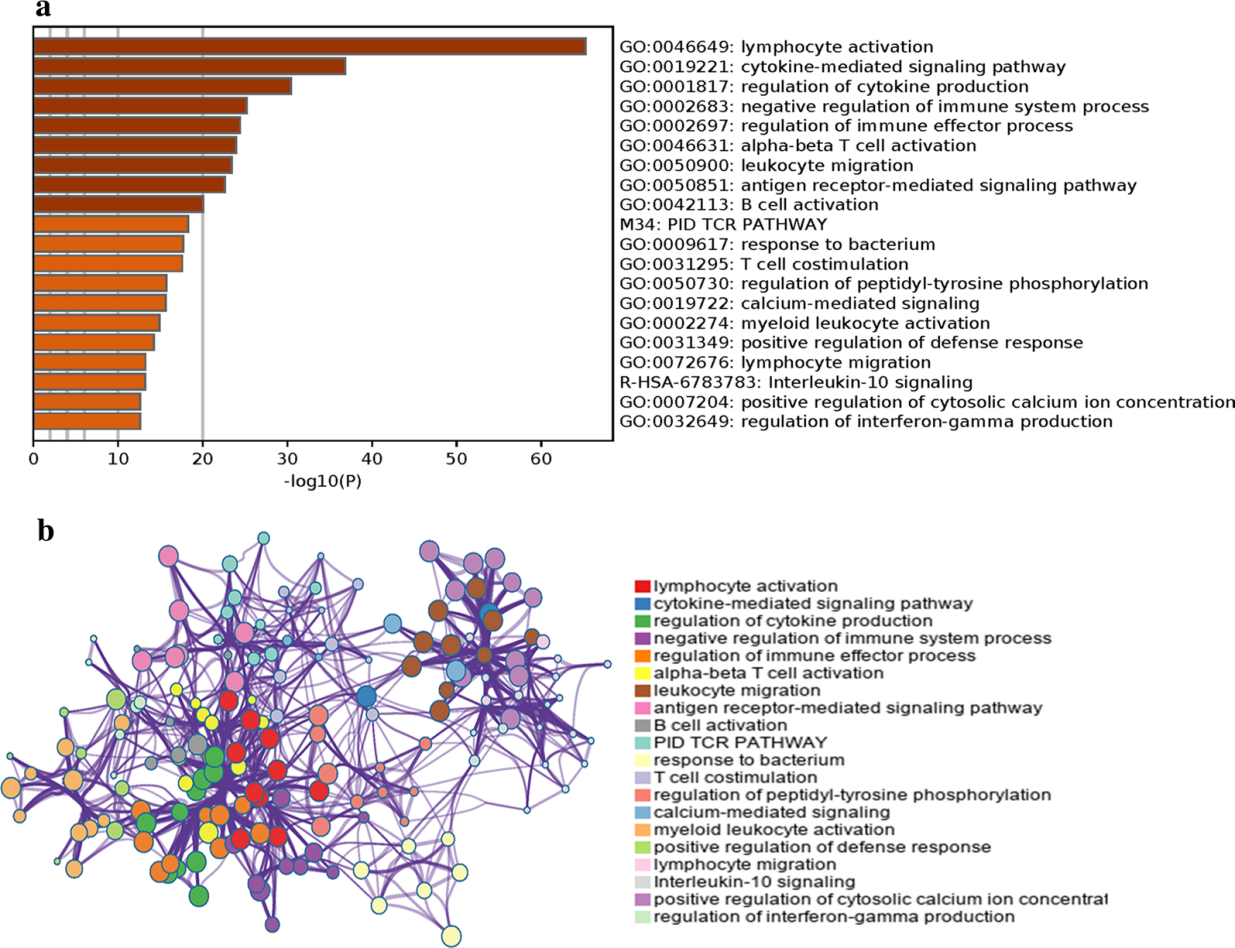
Gene enrichment analysis of the immune-related lncRNAs signature in the Metascape database. **a** The 20 enrichment terms. **b** The network of the enrichment terms

To further explore the potential functional roles of the four IRLs, significantly associated IRGs (Pearson coefficient > 0.7 and *P* < 0.05) were included in pathway enrichment via Metascape. The top 20 highly enriched pathways are displayed in Fig. 6. The IRGs of the four IRLs clustered most significantly in lymphocyte activation, cytokine-mediated signaling pathway, regulation of cytokine production, negative regulation of immune system process, regulation of immune effector process, alpha–beta T cell activation, leukocyte migration, antigen receptor-mediated signaling pathway, B cell activation, TCR pathway, response to bacterium, T cell costimulation, regulation of peptidyl-tyrosine phosphorylation, calcium-mediated signaling, myeloid leukocyte activation, positive regulation of defense response, lymphocyte migration, interleukin-10 signaling, positive regulation of cytosolic calcium ion concentration, and regulation of interferon-gamma production categories. Moreover, according to LncRRIsearch, a web tool used for comprehensive prediction of lncRNA-mRNA interactions [43], the predicted targets of A1BG-AS1 are FKBP10, NFYC, UBR4, CEACAM19, MT-ND4, PKD1P6, and RNA28S5. The predicted target of AC004477.3 is SEH1L.

## Discussion

With the increasing development of clinical management and comprehensive treatment of BC, prognosis of the survival of these patients has greatly improved. Nevertheless, many BC patients experience tumor recurrence [44]. High-throughput biological technologies have provided a platform for exploring the molecular characteristics of different tumors [45]. Many lncRNAs have been used to predict BC prognosis [44, 46, 47], though few IRL signatures have been identified as improving BC survival prediction. Consequently, it is necessary to single out prognostic IRLs integrating TNM stage to predict 5-year RFS of BC patients. In this study, with the combination of four IRLs, TNM stage and tumor subtype, we efficiently constructed and validated a novel IRL-based nomogram to accurately predict 5-year RFS in BC patients. In addition, time-dependent ROC analysis revealed that the IRL-based nomogram had more favorable prognostic accuracy than did TNM stage. Moreover, the DCA indicated that the IRL-based model had better clinical application than TNM stage; our IRL-based tool also exhibited good risk stratification ability to significantly categorize BC patients into high- and low-risk groups.

To date, several prognostic tools have been established to predict survival in BC patients [44, 46–55]. Yao et al. identified five lncRNAs as vital prognostic factors to evaluate BC prognosis [46]. However, these models ignored the prognostic value of IRLs for BC patients. Recent studies have suggested that the immune response plays a critical role in cancer progression and recurrence, and accumulating evidence has revealed that IRLs harbor vital predictive value for survival prognosis. For example, Zhang et al. found that si-lncMALAT1 both suppressed osteosarcoma progression and resulted in self-destruction of cells deep within the tumor. This would enable clearance of osteosarcoma cells from the body by the immune system. Therefore, lncMALAT1 was individually correlated with immune system activity and overall survival [56]. LncRNAs cannot encode proteins but do regulate gene expression at different levels, including through epigenetic, transcriptional or posttranscriptional regulation [57]. In the past decade, this new type of gene regulator, namely, lncRNA, has been associated with tumor development, progression, and prognosis, and many lncRNAs have been found to act as prognostic indicators in various tumors. Moreover, lncRNAs can regulate the immune microenvironment of BC [58]. IRLs can be applied to characterize the infiltration of immune cells in tumors. In our study, the IRL signature developed had a better prognostic value than the IRG signature. Thus, IRLs should be integrated into nomograms to improve prognosis prediction in BC. The survival prediction based on the TNM staging system is not yet satisfactory in clinical practice [59], though it is universally acknowledged that the TNM staging system is beneficial to achieve optimal treatment choice for BC patients. The TNM staging system is based on tumor size, lymph node status and metastasis [60]. Nonetheless, malignant behavior in BC is mainly determined by the molecular characteristics. Thus, incorporating the TNM staging system and molecular biomarkers is indispensable for developing an accurate tool for prognostic assessment in BC patients [61].

Some limitations should be noted in the present study. First, TCGA does not include postoperative treatment information (chemotherapy and hormone therapy). Therefore, we are unable to screen low-risk patients to tailor adjuvant treatment. Second, the molecular mechanism of the IRLs should be further explored by additional experiments. Third, our established model should be verified by prospective, large-scale multicenter datasets.

## Conclusions

In summary, we identified IRLs signatures significantly associated with the 5-year RFS of BC and efficiently constructed a novel prognostic model for 5-year RFS prediction by incorporating four IRLs, TNM stage and tumor subtype. Our model can improve the predictive power of the AJCC TNM staging system and confirm high-risk patients with tumor recurrence to receive appropriate treatment.

## Data Availability

All data was obtained from The Cancer Genome Atlas (TCGA, https://tcgadatanci.nih.gov/tcga/).

## Abbreviations

IRLs: Immune-related lncRNAs
BC: Breast cancer
TCGA: The Cancer Genome Atlas
RFS: Recurrence-free survival
DCA: Decision curve analysis
AJCC: American Joint Committee on Cancer
TNM: Tumor-node-metastasis
lncRNAs: Long non-coding RNAs
ER: Estrogen receptor
PR: Progesterone receptor
HER2: Human epithelial growth factor receptor 2
GDPH: Guangdong Provincial People’s Hospital
IRGs: Immune-related genes
MSDB: Molecular Signatures Database
LASSO: Least absolute shrinkage and selection operator
CPHRA: Cox proportional hazards regression analysis
AUC: Area under the curve
ROC: Receiver operating characteristic
